# Machine Learning Efficiency in Predicting Obstructive Coronary Artery Disease in Patients with Non-ST Elevation Acute Coronary Syndrome in the First Hours of Admission

**DOI:** 10.17691/stm2025.17.3.05

**Published:** 2025-06-30

**Authors:** M.M. Tsivanyuk, K.I. Shakhgeldyan, M.A. Markov, V.G. Shirobokov, B.I. Geltser

**Affiliations:** Senior Researcher, Laboratory of Big Data Analysis in Healthcare and Medicine; Far East Federal University, 10 Ayaks Village, Russkiy Island, Vladivostok, 690922, Russia; Interventional Cardiologist; Vladivostok City Clinical Hospital No.1, 22 Sadovaya St., Vladivostok, 690078, Russia; Associate Professor, Head of the Laboratory of Big Data Analysis in Healthcare and Medicine; Far East Federal University, 10 Ayaks Village, Russkiy Island, Vladivostok, 690922, Russia; Director of Scientific and Educational Center for Artificial Intelligence; Vladivostok State University, 41 Gogolya St., Vladivostok, 690014, Russia; Master’s Student, Scientific and Educational Center for Artificial Intelligence; Vladivostok State University, 41 Gogolya St., Vladivostok, 690014, Russia; Junior Researcher, Scientific and Educational Center for Artificial Intelligence; Vladivostok State University, 41 Gogolya St., Vladivostok, 690014, Russia; Professor, Corresponding Member of Russian Academy of Sciences, Deputy Director of School of Medicine and Life Sciences; Far East Federal University, 10 Ayaks Village, Russkiy Island, Vladivostok, 690922, Russia

**Keywords:** coronary arteries, obstructive disease, acute coronary syndrome, unstable angina, prognostic models, risk stratification, stochastic gradient boosting

## Abstract

**The aim of the study** was to assess the accuracy of prognostic models for obstructive coronary artery disease (OCAD) in the first hours of admission in patients with non-ST segment elevation acute coronary syndrome (NSTE-ACS).

**Materials and Methods:**

The study involved 610 patients with low- and intermediate-risk NSTE-ACS (Me — 62 years). Based on invasive coronary angiography findings the patients were divided into 2 groups: the first — 363 (59.5%) patients with OCAD (coronary artery luminal occlusion ≥50%), the second — 247 (40.5%) patients without coronary obstruction (<50%). Clinical and functional status was assessed using 62 parameters available at the early hospitalization including: clinical and demographic, anthropometric, laboratory, electrocardiographic and echocardiographic data.

OCAD predictive models were developed using machine learning methods: multifactorial logistic regression, random forest, and stochastic gradient boosting (SGB). The models contained the sets of predictors identified during the initial medical examination in the hospital (the first scenario), after 1-hour observation (the second scenario), and 3 h later (the third scenario). The quality of the models was assessed using six metrics. The impact degree of individual predictors on the study endpoint was determined by the Shapley method of additive explanation (SHAP). OCAD probability stratification was performed by distinguishing the categories of low, medium, high and very high risk.

**Results:**

Based on machine learning methods, OCAD predictive models were developed, among which the best quality metrics were demonstrated by SGB models with the sets of predictors corresponding to three prognostic scenarios (the area under ROC curve: 0.846, 0.887, and 0.949, respectively). Using the SHAP method, we identified the factors with a dominant impact on OCAD, which included the anthropometric indicators (waist circumference, hip circumference, and their ratio) — in the first and second prognostic scenarios; and global longitudinal systolic strain of the left ventricle — in the third scenario. Based on SGB model data there were distinguished the categories of low, medium, high and very high risk of OCAD, their digital ranges depended on the prognostic scenarios.

**Conclusion:**

The prognostic OCAD models developed based on SGB enable to highly accurately assess the degree of coronary damage in NSTE-ACS patients in the first hours of hospitalization. The highest accuracy of OCAD prediction was demonstrated by the models of the third scenario, the structure of which, in addition to anamnestic, anthropometric and ECG data, included clinical and biochemical blood parameters and echocardiographic indicators. Thus, OCAD risk stratification using the mentioned models can be a useful tool in selecting the optimal myocardial revascularization strategy.

## Introduction

Coronary artery disease (CAD) ranks the third leading position in the structure of cardiovascular diseases (CVD) in most countries in the world. The half of CAD cases debut as acute coronary syndrome (ACS), which is the most frequent reason for urgent hospitalization. NonST elevation ACS (NSTE-ACS) occurs more frequently than ACS with ST elevation, and its development is associated with a wider range of pathophysiological mechanisms [1]. Alongside with incomplete occlusion of epicardial arteries, NSTE-ACS causes include spasm or spontaneous dissection of coronary arteries, myocardial muscular bridging, microvascular spasm, tako-tsubo syndrome, etc. [2].

Over the last years, based on invasive coronary angiography (CAG) findings, the cases of non-obstructive coronary artery disease (NOCAD) have been increasingly frequently recorded: 25% of NSTE-ACS patients appear to have NOCAD [3]. In Russia, in an annual increase in applying CAG, approximately in 50% of patients the procedure fails to result in myocardial revascularization, mostly, due to the absence of obstructive coronary artery disease (OCAD). Therefore, the problems related to the assessment of CAG applicability in low and intermediate NSTE-ACS risk have been debated a lot that is due to the necessity to limit its unreasonable application, the reduction of unreasonable complications of an invasive procedure and irrational expenses in public health care. The relevance of the approach is proved by comparable results of hospital prognosis in NSTE-ACS patients with low risk of unfavorable outcome in using multi-spiral computed tomography of coronary arteries and invasive CAG [4]. A promising tool of noninvasive OCAD verification are prognostic algorithms based on current techniques of machine learning enable to highly accurately assess the coronary artery disease degree [3]. However, the reliability of such algorithms can depend on implementation lead time after NSTE-ACS patients are admitted to a hospital, since a set of predictors included into a model can influence the prognosis accuracy.

**The aim of the study** was to assess the accuracy of prognostic OCAD models in the first hours of admission in patients with NSTE-ACS.

## Materials and Methods

A one-center retrospective study was carried out based on the data on 610 patients with NSTE-ACS aged from 30 to 80 years (age median (Me) — 62 years, 95% confidence interval (CI) — 61–63) admitted to Emergence Cardiac Care Unit in Vladivostok Clinical Hospital No.1 in 2021–2024. The risk assessment was calculated according to GRACE: 457 patients (75%) had low risk, 153 patients (25%) — intermediate risk. The data were collected in accordance with Good Clinical Practice standards and Helsinki Declaration principles (2024). The study protocol was approved by the Ethics Committee by Far East Federal University (protocol No.4 dated December 5, 2019). All patients gave their written consent.

The inclusion criteria were the following: NSTE-ACS patients without proved myocardial necrosis according to highly-sensitive troponin I, who underwent invasive CAG.

The non-inclusion criteria were: acute myocardial infarction, percutaneous coronary intervention or coronary bypass in past medical history. According to invasive CAG, the patients were divided into 2 groups: group 1 included 363 OCAD patients (59.5%) (coronary artery lumen narrowing ≥50%), group 2 — 247 patients (40.5%) with NOCAD (<50%).

The patients’ clinical and functional status was assessed by 62 signs including clinical and demographic, anthropometric (waist circumference — WC, hip circumference — HC, and their ratio — WC/ HC), laboratory, electrocardiographic (ECG) and echocardiographic (echoCG) data. Venous blood was drawn in patients admitted to hospital followed by blood examination using the analyzers Mindray BS- 800M (Mindray, China) and Sysmex XN-550 (Sysmex Corporation, Japan). We determined hemoglobin (Hb), red blood cells (RBC), white blood cells (WBC), platelets (PLT), RBC sedimentation rate, hematocrit (HCT), plateletcrit (PCT), mean cell volume (MCV) of RBC, mean cell Hb in RBC (MCH), mean cell Hb concentration in erythrocytes (MCHC), RBC distribution width (RDW- CV), mean PLT volume (MPV), PLT distribution width (PDW), lipid profile characteristics, glucose, uric acid (UA), C-reactive protein (CRP), and creatinine concentration.

According to ECG findings we assessed the duration of PQ, QT, QRS intervals; the presence of ST segment depression ≥0.1 mV in two and more neighboring leads, ST elevation ≥0.1 mV in aVR lead. EchoCG was performed using GE Vivid S60 (GE Healthcare, USA) according to an established procedure. There were determined the interventricular septum thickness, left ventricular (LV) posterior wall thickness, LV myocardial mass index. We recorded LV end diastolic volume and LV systolic volume, the left atrial volume. There were calculated LV systolic output and ejection fraction. To determine LV global longitudinal systolic strain (GLSS) we used the data on apical 4-chamber position of tissue Doppler myocardial images using the software of GE Vivid S60.

The study endpoint was represented as OCAD in the form of a binary sign (“absence” or “presence”). Input signs (a subgroup of potential predictors) were expressed in the form of continuous and categorical variables.

### Statistical processing and machine learning methods

Data distribution in the text according to Kolmogorov–Smirnov test differed from normal, and for that reason nonparametric statistical methods were used. The indicators were represented by the median (Me) and interquartile ranges [Q1; Q3], for intergroup comparison of continuous variables we used Mann–Whitney test, while for categorical variables — χ^2^. Odds ratio (OR) and 95% CI were calculated using Fisher exact test. Differences were considered significant when p<0.05. To develop prognostic OCAD models, we used machine learning methods: multifactorial logistic regression (MLR), random forest (RF), and stochastic gradient boosting (SGB). The models were developed on a learning sample (9/10) of patients and verified on a test sample (1/10). The quality of models was assessed by six metrics: the area under ROC curve (AUC), F-measure, sensitivity (Se), specificity (Sp), positive (PPV), and negative (NPV) prognostic value.

The study design involved 4 stages. The first stage involved the intergroup analysis of clinical and functional factors to distinguish potential predictors linearly related to OCAD. The second stage was devoted to the development of prognostic OCAD models with the sets of predictors obtained through an initial physical examination in hospital, an hour and 3 h after admission, it corresponding to the time intervals of risk stratification of unfavorable NSTE-ACS outcome according to the study findings of cardiac troponin level [1]. The third using Shapley method of additive explanation (SHAP) determined the effect intensity of certain predictors of prognostic OCAD models on the study endpoint. The fourth stage involved OCAD risk stratification by SGB model data in accordance with the algorithm, which included the division of the dataset into 5 subsamples using stratified k-fold followed by training the models in a cycle on four of these samples and testing on one sample by keeping records of the prognostic results. To make a decision on OCAD presence, we tried a cutoff threshold of the calculation probability in such a way that Se and Sp values were as close as possible. The cases referred to OCAD were those when the calculation probability exceeded the cutoff threshold, and those referred to NOCAD had the calculated probability, which did not exceed the cutoff threshold. The probability range lower than the cutoff threshold was divided into 2 levels — low and intermediate OCAD risk, and that higher the threshold — into high and very high risk. For division we used the median values of predicted probabilities in comparison groups, which were compared with the low- and very high-risk boundaries. The risk stratification accuracy was assessed using the metric Accuracy (Ac). The data were analyzed and the models were developed using Python language with an open-source code, v. 3.9.16.

## Results

***The first study stage*** established the statistical significance of the differences by 36 indicators of clinical and functional status of NSTE-ACS patients according to the intergroup analysis findings (Table 1). The maximal significant level was associated with gender identity (male), smoking status, CVD in family history, the indicators: WC, WC/HC, RDW-CV, MPV, PDW; the levels of total cholesterol, high density lipoprotein cholesterol (HDL CH) and low-density lipoprotein cholesterol (LDL CH), creatinine, CRP, UA, and LV GLSS (p<0.0001). The highest OR was recorded in the presence of chronic obstructive pulmonary disease (OR=8.41), while the less marked though statistically significant OCAD probability was associated with tobacco smoking (OR=2.6), CVD in family history (OR=2.3), male gender (OR=2.2) and diabetes mellitus comorbidity (OR=1.67). In addition, arterial hypertension and atrial fibrillation in the comparison groups were recorded equifrequently, and it was the evidence of no linear relations of these factors with OCAD (OR=0.91, p=0.8 and OR=0.86, p=0.7, respectively). According to the intergroup statistical analysis, the indicators of age, height, body mass of the patients under study, the concentrations of glucose and total protein in blood, systolic and diastolic arterial pressure were comparable values indicating the low probability of their effect on OCAD.

**T a b l e 1:** Clinical and functional indicators of patients with non-ST elevation acute coronary syndrome, Мe [Q1; Q3]

Indicator	Group 1 (OCAD), n=363	Group 2 (NOCAD), n=247	OR (95% CI)	^p^
***Clinical and demographic indicators***				
Male (n/%)	252/69.4	127/51.4	2.2 (1.5–3.0)	<0.0001
Age (years)	62 [56; 69]	62 [54; 68]	—	0.16
Smokers (n/%)	149/41.0	52/21.1	2.6 (1.8–3.8)	<0.0001
Negative family history in CVD (n/%)	108/29.75	38/15.38	2.3 (1.5–3.5)	<0.0001
AH (n/%)	312/86	215/87	0.91 (0.57–1.46)	0.8
COPD (n/%)	12/3.3	1/0.4	8.41 (1.09–65.1)	0.0032
DM (n/%)	66/18.2	29/11.7	1.67 (1.04–2.67)	0.04
AF (n/%)	28/7.7	22/8.9	0.86 (0.48–1.53)	0.7
SAP (mm Hg)	140 [130; 160]	140 [130; 150]	—	0.12
DAP (mm Hg)	80 [80; 90]	80 [80; 90]	—	0.96
***Anthropometric indicators***				
Height (cm)	170 [164; 176]	170 [164; 176]	—	0.48
Body mass (kg)	82.0 [73.5; 92.0]	84.0 [75.5; 92.0]	—	0.68
BMI	28.0 [25.6; 31.1]	28.60 [26.10; 31.75]	—	0.3
WC (cm)	97 [91; 102]	93 [88; 101]	—	<0.0001
HC (cm)	100 [96; 106]	103 [98; 108]	—	0.004
WC/HC	0.96 [0.90; 1.00]	0.90 [0.86; 0.94]	—	<0.0001
***Full blood count indicators***				
Hb (g/L)	144 [132; 154]	144 [133; 154]	—	0.6
WBC (×10^9^/L)	7.9 [6.5; 9.6]	7.5 [6.2; 8.8]	—	0.0006
RBC (×10^9^/L)	4.59 [4.24; 4.94]	4.65 [4.29; 4.98]	—	0.4
PLT (×10^9^/L)	239 [200; 281]	238 [198; 274]	—	0.46
ESR (mm/h)	10 [5; 16]	8 [4; 12]	—	0.0014
HCT (%)	42.2 [39.7; 44.5]	42.3 [40.1; 44.5]	—	0.795
MCV (fl)	91.50 [88.95; 94.20]	90.70 [89.50; 92.18]	—	0.019
MCH (pg)	31.40 [30.25; 33.0]	31.5 [30.2; 32.6]	—	0.46
MCHC (g/L)	344 [336; 351]	345 [336; 354]	—	0.25
RDW-CV (%)	14.7 [14.1; 15.2]	14.3 [14.0; 14.7]	—	<0.0001
MPV (fl)	8.6 [8.1; 9.0]	8.8 [8.5; 9.1]	—	<0.0001
PDW (%)	15.7 [15.4; 15.8]	15.4 [14.9; 15.8]	—	<0.0001
PCT (%)	0.21 [0.19; 0.24]	0.21 [0.19; 0.23]	—	0.08
***Biochemical indicators***				
Glucose (mmol/L)	5.9 [5.4; 6.8]	5.9 [5.4; 6.6]	—	0.97
Creatinine (μmol/L)	89 [78; 102]	80.5 [70.0; 94.0]	—	<0.0001
GFR (ml/min/1.73 m^2^)	73.3 [61.5; 85.6]	77.20 [65.20; 92.98]	—	0.0098
TC (mmol/L)	5.70 [4.76; 6.50]	5.21 [4.45; 5.85]	—	<0.0001
TG (mmol/L)	1.42 [1.08; 2.15]	1.3 [0.9; 1.9]	—	0.018
HDL CH (mmol/L)	1.20 [1.01; 1.49]	1.34 [1.15; 1.69]	—	<0.0001
LDL CH (mmol/L)	3.6 [2.8; 4.3]	3.1 [2.4; 3.7]	—	<0.0001
ALT (U/L)	25 [17; 33]	27 [19; 39]	—	0.02
AST (U/L)	23 [19; 32]	23 [19; 31]	—	0.96
CRP (mg/L)	2.0 [0.1; 5.5]	0.9 [0; 3.7]	—	<0.0001
Total protein (g/L)	74 [70; 78]	74 [71; 78]	—	0.4
UA (μmol/L)	375 [349; 418]	320 [279; 381]	—	<0.0001
PTT (s)	13.1 [11.5; 14.7]	12.65 [11.40; 14.50]	—	0.3
Fibrinogen (g/L)	3.12 [2.66; 3.70]	2.91 [2.40; 3.45]	—	0.00056
aPPT (s)	29.8 [26.3; 34.4]	28.35 [25.40; 32.40]	—	0.0016
***Electrocardiographic indicators***				
HR per min	67 [60; 75]	68 [62; 75]	—	0.699
ST depression (n/%)	93/25.6	52/21.1	1.27 (0.86–1.87)	0.26
Т inversion (n/%)	174/29.5	66/26.7	1.13 (0.79–1.62)	0.56
QT (s)	0.40 [0.37; 0.42]	0.40 [0.37; 0.42]	—	0.4
QRS (s)	0.093 [0.080; 0.100]	0.09 [0.08; 0.10]	—	0.4
PQ (s)	0.161 [0.160; 0.190]	0.16 [0.14; 0.18]	—	0.00036
ST_aVR_ elevation (n/%)	146/40.2	61/24.7	2.0 (1.4–2.9)	0.00018
***Echocardiographic indicators***				
IVS (mm)	11 [10; 12]	10 [9; 12]	—	0.0127
LV PW (mm)	10 [9; 12]	10 [9; 11]	—	0.055
LA volume (ml)	47 [40; 58]	46 [38; 56]	—	0.2
RA volume (ml)	42 [34; 50]	40 [34; 47]	—	0.0115
EDD (mm)	50 [45; 53]	48 [45; 52]	—	0.07
EDV (ml)	111 [89; 134]	103 [89; 124]	—	0.019
ESV (ml)	40 [30; 52]	36 [28; 44]	—	0.0014
LV EF (%)	64 [57; 70]	65 [61; 70]	—	0.18
PG AoV (mm Hg)	6.1 [4.2; 8.0]	6.80 [4.98; 8.0]	—	0.03
LV MMI (g/m^2^)	115.7 [94.5; 138.2]	106.3 [90.5; 126.4]	—	0.0007
LV GLSS (%)	18.1 [16; 19]	20.2 [19.5; 21.5]	—	<0.0001

H e r e: AH — arterial hypertension; AF — atrial fibrillation; ALT — alanine aminotransferase; aPPT — activated partial thromboplastin time; AST — aspartate aminotransferase; BMI — body mass index; COPD — chronic obstructive pulmonary disease; CRP — С-reactive protein; CVD — cardiovascular diseases; DAP — diastolic arterial pressure; DM — diabetes mellitus; EDD — end diastolic diameter; EDV — end diastolic volume; EF — ejection fraction; ESR — erythrocyte sedimentation rate; ESV — end systolic volume; GFR — glomerular filtration rate; GLSS — global longitudinal systolic strain; Hb — hemoglobin; HC — hip circumference; HCT — hematocrit; HDL CH — high-density lipoprotein cholesterol; HR — heart rate; IVS — intraventricular septum; LA — left atrium; LDL CH — low-density lipoprotein cholesterol; LV — left ventricle; MCH — mean cell hemoglobin; MCHC — mean cell hemoglobin concentration; MCV — mean cell volume; MMI — myocardial mass index; MPV — mean platelet volume; NOCAD — non-obstructive coronary artery disease; OCAD — obstructive coronary artery disease; PCT — plateletcrit; PDW — platelet distribution width; PG AoV — pressure gradient at aortic valve; PLT — platelets; PTT — prothrombin time; PW — posterior wall; RA — right atrium; RBC — red blood cells; RDW-CV — red cell distribution width, coefficient of variation; SAP — systolic arterial pressure; TC — total cholesterol; TG — triglycerides; UA — uric acid; WBC — white blood cells; WC — waist circumference.

***The second study stage*** using MLR, RF, and SGB concerned the development of prognostic models enable to assess OCAD probability prior to performing invasive CAG. The predictors selected from the data set, which were obtained on an initial examination, when the patients were admitted to hospital, included the following indicators: male gender, smoking status, negative family history, body mass, height, WC, HC, PQ interval, the presence of ST elevation in aVR lead. An hour after the patient’s admission the basic list of predictors was added by the full blood count findings (WBC, RDW-CV, PDW, MPV), and 3 h later — by biochemical and echo-CG data (LDL CH, UA, and LV GLSS). The analysis of quality metrics showed the models developed on different time intervals using SGB method had higher prognostic accuracy compared with the models developed on the basis of MLR and RF (Tables 2–4). It is worth noting that the successive range expansion of predictors was accompanied by the prognosis quality increase, the indicators of which achieved their maximum values when using the total complex of clinical and functional data available for analysis (see Table 4).

**T a b l e 2:** Prognostic models of obstructive coronary artery disease with the predictors obtained in patients with acute coronary syndrome with non-elevation of segment on initial medical examination when admitted to hospital, Me [Q1; Q3]

Metrics	Cross-validation			Final testing		
	MLR	SGB	RF	MLR	SGB	RF
AUC	0.816 [0.814; 0.818]	0.834 [0.836; 0.841]	0.824 [0.821; 0.826]	0.814 [0.808; 0.821]	0.846 [0.840; 0.853]	0.828 [0.82; 0.836]
Se	0.746 [0.744; 0.749]	0.760 [0.757; 0.763]	0.734 [0.730; 0.738]	0.736 [0.726; 0.746]	0.763 [0.753; 0.772]	0.742 [0.732; 0.751]
Sp	0.745 [0.742; 0.748]	0.765 [0.776; 0.769]	0.749 [0.745; 0.754]	0.746 [0.734; 0.758]	0.771 [0.758; 0.784]	0.754 [0.742; 0.767]
PPV	0.817 [0.816; 0.819]	0.832 [0.829; 0.835]	0.818 [0.815; 0.821]	0.811 [0.804; 0.818]	0.832 [0.824; 0.84]	0.817 [0.809; 0.825]
NPV	0.668 [0.665; 0.671]	0.685 [0.682; 0.688]	0.657 [0.653; 0.661]	0.660 [0.652; 0.668]	0.691 [0.682; 0.699]	0.667 [0.658; 0.676]
F-measure	0.778 [0.775; 0.780]	0.791 [0.789; 0.794]	0.771 [0.768; 0.774]	0.771 [0.764; 0.777]	0.795 [0.788; 0.801]	0.777 [0.770; 0.784]

N o t e. The model predictors: male gender, body mass, height, WC, HC, WC/HC, smoking, PQ interval, ST elevation in aVR lead, negative family history.

**T a b l e 3:** Prognostic models of obstructive coronary artery disease with the predictors obtained in patients with acute coronary syndrome with non-elevation of segment an hour after admission to hospital, Me [Q1; Q3]

Metrics	Cross-validation			Final testing		
	MLR	SGB	RF	MLR	SGB	RF
AUC	0.883 [0.882; 0.885]	0.946 [0.944; 0.947]	0.937 [0.936; 0.938]	0.881 [0.875; 0.887]	0.949 [0.946; 0.953]	0.940 [0.936; 0.944]
Se	0.814 [0.811; 0.816]	0.866 [0.864; 0.868]	0.854 [0.852; 0.856]	0.811 [0.803; 0.820]	0.872 [0.865; 0.880]	0.861 [0.854; 0.869]
Sp	0.803 [0.900; 0.806]	0.871 [0.867; 0.874]	0.867 [0.865; 0.87]	0.80 [0.78; 0.81]	0.874 [0.863; 0.884]	0.865 [0.856; 0.874]
PPV	0.862 [0.860; 0.864]	0.911 [0.909; 0.913]	0.908 [0.908; 0.909]	0.860 [0.854; 0.866]	0.913 [0.907; 0.919]	0.906 [0.900; 0.912]
NPV	0.750 [0.747; 0.753]	0.820 [0.817; 0.823]	0.806 [0.803; 0.808]	0.741 [0.732; 0.750]	0.822 [0.813; 0.831]	0.808 [0.799; 0.816]
F-measure	0.835 [0.833; 0.837]	0.886 [0.885; 0.888]	0.878 [0.877; 0.880]	0.834 [0.829; 0.840]	0.891 [0.886; 0.897]	0.882 [0.878; 0.887]

N o t e. The model predictors: male gender, body mass, WC, HC, WC/HC, smoking, ST elevation in aVR lead, negative family history, PQ interval, WBC, RDW-CV, PDW, MPV.

**T a b l e 4:** Prognostic models of obstructive coronary artery disease with the predictors obtained in patients with acute coronary syndrome with non-elevation of segment 3 h after admission to hospital, Me [Q1; Q3]

Metrics	Cross-validation			Final testing		
	MLR	SGB	RF	MLR	SGB	RF
AUC	0.831 [0.829; 0.833]	0.880 [0.878; 0.882]	0.870 [0.868; 0.872]	0.832 [0.826; 0.837]	0.887 [0.882; 0.891]	0.899 [0.886; 0.912]
Se	0.766 [0.764; 0.768]	0.798 [0.796; 0.801]	0.786 [0.731; 0.789]	0.758 [0.749; 0.767]	0.803 [0.794; 0.811]	0.819 [0.794; 0.845]
Sp	0.761 [0.758; 0.764]	0.800 [0.797; 0.805]	0.789 [0.785; 0.792]	0.765 [0.753; 0.776]	0.812 [0.803; 0.824]	0.804 [0.768; 0.84]
PPV	0.831 [0.829; 0.833]	0.860 [0.858; 0.863]	0.851 [0.849; 0.854]	0.827 [0.820; 0.834]	0.864 [0.858; 0.87]	0.851 [0.843; 0.858]
NPV	0.690 [0.687; 0.693]	0.732 [0.728; 0.735]	0.716 [0.713; 0.719]	0.684 [0.676; 0.692]	0.739 [0.731; 0.747]	0.723 [0.713; 0.732]
F-measure	0.795 [0.793; 0.796]	0.860 [0.824; 0.828]	0.815 [0.813; 0.817]	0.790 [0.784; 0.796]	0.831 [0.826; 0.836]	0.818 [0.812; 0.924]

N o t e. The model predictors: male gender, body mass, WC, HC, WC/HC, smoking, ST elevation in aVR lead, negative family history, PQ, RDW-CV, PDW, MPV, LV GLSS, LDL CH, UA.

**Figure 1.**
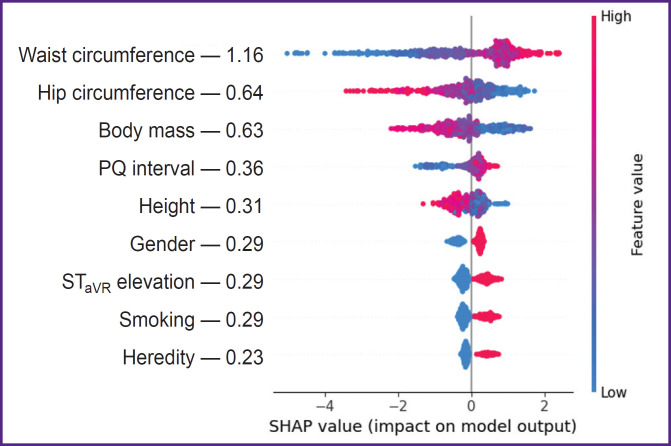
SHAP-diagram of the effect of the predictors of stochastic gradient boosting model obtained on an initial medical examination of acute coronary syndrome patients with non-ST elevation on obstructive coronary artery disease

**Figure 2.**
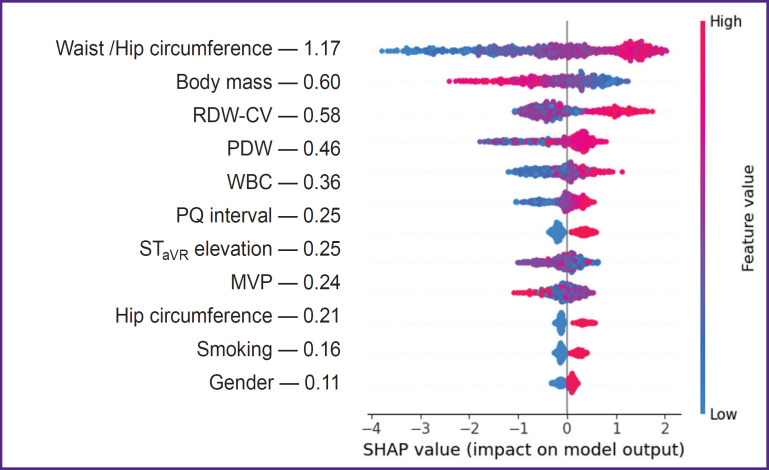
SHAP-diagram of the effect of the predictors of stochastic gradient boosting model obtained 1 h after hospitalization in acute coronary syndrome patients with non-ST elevation on obstructive coronary artery disease

**Figure 3.**
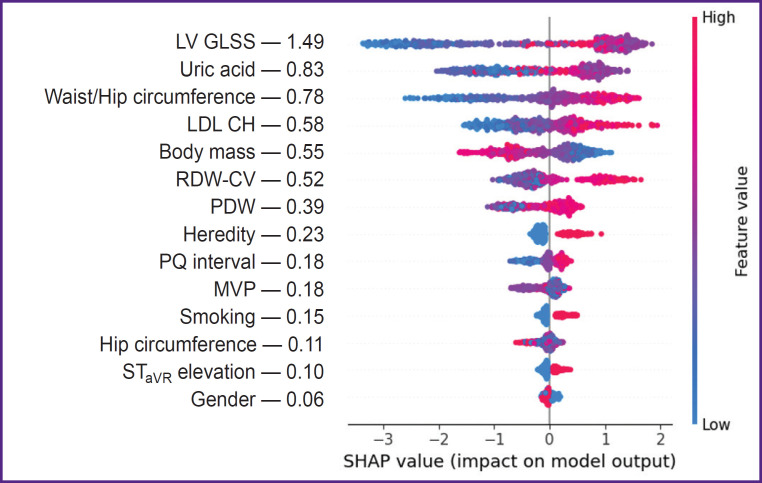
SHAP-diagram of the effect of the predictors of stochastic gradient boosting model obtained 3 h after hospitalization in acute coronary syndrome patients with non-ST elevation on obstructive coronary artery disease

***The third study stage*** using SHAP method determined the effect degree of certain predictors included in the structure of the best SGB models on the endpoint implementation. The analysis findings showed WC to demonstrate the closest association with OCAD among other predictors of prognostic models developed according to the initial medical examination data in the hospital, WC growth being associated with an increasing risk of hemodynamically significant stenoses of coronary arteries (Figure 1). Less pronounced relations with the endpoint were recorded in the indicators of gender identity (male gender), body mass, height, HC, tobacco smoking, PQ interval and ST elevation in aVR lead, while the negative CVD family history was found to have the minimal effect on OCAD. When ranging the predictive value of SGB model predictors including the full blood count indicators we stated the effect intensity of the latter on OCAD to be far below the anthropometric WC/HW index, its level being maximum (Figure 2). The effect on RDW-CV endpoint was comparable with the patients’ body mass, and MPV — with the sign of ST elevation in aVR lead. In the prognostic SGB model, the predictors of which were supplemented by biochemical and echo-CG indicators, LV GLSS demonstrated the highest interaction with OCAD (Figure 3). The following indicators showed the less pronounced effect on the endpoint: UA, WC/HC, LDL CH, and body mass, and the minimal effect was demonstrated by the factors: male gender and tobacco smoking.

***The fourth study stage**,* according to SGB model data including different sets of predictors, stratified OCAD risk corresponding to time prediction intervals (Table 5). OCAD probability was checked against distinguishing the categories of low, intermediate, high and very high risk, their digital range depended on the structure of the models. The study findings showed the prognostic model of scenario 1 developed on the basis of the predictors obtained on the initial medical examination on admission to hospital enable to select OCAD patients from the high-risk group with the accuracy according to Ac metric equal to 92.6%. In accordance with the model data, in the low OCAD risk patients, the prognosis accuracy of hemodynamically insignificant stenosis was slightly lower (Ac — 86.6%). The models of the second and third scenarios, their structure was supplemented by hematological and echoCG predictors, had the higher accuracy of identifying OCAD patients in very high risk of coronary stenosis (Ac — 95.0–99.4%) and NOCAD patients — in its low risk (Ac — 96.8–100%). Moreover, OCAD prognosis quality among the patients with intermediate and high risk in all modeling scenarios was significantly lower (Ac — 71.2–78.8%).

**T a b l e 5:** Obstructive coronary artery disease (OCAD) risk stratification in patients with acute coronary syndrome with non-ST elevation (%)

Risk	Scenario 1		Scenario 2		Scenario 3	
	OCAD probability ranges	Accuracy	OCAD probability ranges	Accuracy	OCAD probability ranges	Accuracy
Low	<52	86.6	<7	100	<10	96.8
Intermediate	52–79	52.9	7–77	71.2	10–65	75.2
High	79–92	69.7	77–95	75.5	65–98	78.8
Very high	>92	92.6	>95	95.7	>98	99.4

## Discussion

The extended application of ‘classical’ scales in clinical practice to assess unfavorable outcome risks (GRACE, HEART, TIMI) in different ACS forms has enabled to enhance the quality of the medical care provided and reduce decision-making time regarding invasive myocardial revascularization. NSTE-ACS is one of the most intricate CAD forms, and it is due to a number of factors: heterogeneous population of patients characterized by different clinical forms of the disease; ambiguousness of approaches to risk stratification and assessment of its efficiency; the difficulty in choosing optimal treatment strategies that is mostly related to a multivessel disease of coronary arteries, patients’ elderly and old age [5]. In the present study, according to dynamic ECG and troponin I control, all patients were diagnosed to have unstable angina, its clinical presentation referred to not high risk of unfavorable outcomes by GRACE. According to CAG data, nearly 60% patients of the cohort under study were found to have OCAD, and 40% — NOCAD. On the one hand, it confirms the well-known concept of heterogeneity of pathophysiological mechanisms participating in developing acute myocardial ischemia in unstable angina, and on the other hand, it is the evidence of the practicability of using current prognostic technologies to assess the coronary artery damage degree in this category patients prior to an invasive procedure.

Previously [5, 6], it was demonstrated that the presence of the preliminary information on the coronary blood flow in NSTE-ACS patients obtained through the machine learning algorithms can enhance the prognostic resource of risk assessing scales contributing to choosing optimal treatment strategies. The significance of the approach can be demonstrated by an example of GRACE scale, its insufficient efficiency some researchers have associated with the lack of the data on the following indicators in its structure: patients’ gender identity, comorbidity, and excessive dependence of generated conclusion decisions on an age indicator and others. The scale modified by extending the predictors’ range exceeded the accuracy of hospital lethality risk stratification in different clinical ACS forms [7]. Additionally, the possibilities for improving riskometry tools by means of noninvasive OCAD indicators are currently limited due to the lack of the generally accepted prognostic algorithms of coronary obstruction [8]. To solve the problem, in the present study for the first time there were developed the prognostic OCAD models in patients with unstable angina based on the predictors obtained within the first hours of admission, and corresponding to time intervals, which are stated by the clinical recommendations for early risk stratification of unfavorable outcomes in NSTE-ACS using high- sensitivity troponins [1, 2].

The present study findings showed the first scenario models to have good OCAD prognosis quality so far, the highest predictive potential was demonstrated by 3 anthropometric indicators characterizing patients’ metabolic status: WC, HC, and body mass (see Table 2 and Figure 1). Abdominal obesity is referred to the most ‘aggressive’ factors of cardiometabolic risk, coronary arteries being their main targets. It is confirmed by the predictive value of WC/HC≥0.9 and WC/height≥0.69 ratios in the prognostic OCAD model developed by an ensemble method based on 10 MLR algorithms (AUC — 0.82) [9]. In the present study the use of these indicators as isolated characteristics in the continuous form enabled to achieve the higher accuracy of OCAD prognosis in the first scenario CGB model (АUC — 0.846).

The prognostic models of the second scenario showed WC/HC index to have a dominant impact on OCAD, while the less pronounced relation with the endpoint was associated with body mass and full blood count indicators (RDW-CV, PDW, and WBC), which are increasingly frequently used for prognostic studies in clinical medicine. In recent years, a number of studied have marked the importance of RDW-CV as an independent indicator of unfavorable outcomes in different diseases including CAD [10, 11]. This characteristic is supposed to be an indicator of oxidative stress intensity, subclinical inflammation and the neuroendocrine system hyperactivity. Some previous studies [12] demonstrated CAD patients with OCAD to have the higher RDW-CV levels than CAD patients with NOCAD (14.59±1.04% vs. 13.60±0.68%, p<0.0001). In the present work, the predictive value of RDW-CV was identified if it was over 14.3%, in the prognostic OCAD model (AUC=0.793, PPV=85.42%). In the study by Namazi et al. [10] RDW-CV level in OCAD patients with a stable CAD course was significantly higher compared with healthy people. In the present study the predictive RDW-CV value regarding OCAD was proved on a cohort of NSTE-ACS patients that specifies the concept of prognostic resource of the factor. PDW, WBC, and MPV indicators had less intensive relations with the study endpoint than RDW-CV (see Figure 2). However, there have appeared more reports, which refer plateletleukocyte ratios to pathophysiological determinants of forming blood clots, inflammation and atherogenesis playing the key role in CAD development [13, 14]. Over 17% increase in PDW level was shown to be associated with more marked coronary obstruction and unfavorable ACS outcomes [15]. In the study by Vogiatzis et al. [16] in ACS patients MPV indicators were found to have the stronger interrelation with SYNTAX scale (Sx) characterizing the severity of coronary blood flow disease. In intact coronary arteries (Sx — 0 scores) the mean MPV value was 9.57 fl, while in Sx at the level of 1–22 scores — 10.6 fl. In patients with Sx in the range of 23–32 scores and over 33 scores, the mean MPV valued increased up to 11.18 and 12.57 fl, respectively.

The prognostic OCAD models of the third scenario developed on the basis of the summarized data of an initial medical examination, full blood count, biochemical blood assay and echo-CG had higher quality metrics than the models of the second and third scenarios that was provided by the predictive potential of new factors: LV GLSS, UA, and LDL CH (see Table 4). In some studies, LV GLSS was recognized as an informative indicator of OCAD. The prognostic properties of the indicator were recorded at the level of less 19% [17, 18]. Hyperuricemia is a proved factor of cardiometabolic risk closely associated with the atherosclerotic remodeling of coronary arteries. OCAD probability was found to increase by 2.2 times, particularly, in UA concentration of over 356 μmol/L [9]. In the present study the effect intensity of UA on OCAD prognosis in the best SGB model was by 1.8 times lower than LV GLSS, but significantly higher than that of the other predictors of the same model (see Figure 3). It is worth noting that the predictive value of LDL CH ranked below WC/HC ratio, although it was comparable with patients’ body mass. OCAD risk stratification in NSTE-ACS patients during the first hours of admission to hospital showed that its accuracy depended on the prognosis scenario, and increased due to the extending range of the predictors of prognostic models (see Table 5). Such approach enables to obtain the preliminary data on the coronary bed condition, and the information can be useful for making a decision on patients’ treatment strategy.

The limitations of the present study are related to its retrospectivity and the necessity to validate the models on NSTE-ACS patients from other healthcare facilities.

## Conclusion

The present study concerns the prognostic OCAD models developed based on SGB, the models enable to highly accurately assess the degree of coronary damage in NSTE-ACS patients in the first hours of hospitalization.

The highest accuracy of OCAD prediction was demonstrated by the models, the structure of which, in addition to anamnestic, anthropometric and ECG data, included clinical and biochemical blood indicators and echo-CG findings. The results of OCAD prognosis and its risk stratification supplement the information on NSTE- ACS patients’ functional status, and can be used when choosing the myocardial revascularization strategy. The prospects for further studies on the problem are related to the testing of the developed prognostic OCAD models as the additional tools to assess the risks of developing adverse events in different clinical ACS forms.

## References

[1] Barbarash O.L., Duplyakov D.V., Zateischikov D.A., Panchenko E.P., Shakhnovich R.M., Yavelov I.S., Yakovlev A.N., Abugov S.A., Alekyan B.G., Arkhipov M.V., Vasilieva E.Yu., Galyavich A.S., Ganyukov V.I., Gilyarevskyi S.R., Golubev E.P., Golukhova E.Z., Gratsiansky N.A., Karpov Yu.A., Kosmacheva E.D., Lopatin Yu.M., Markov V.A., Nikulina N.N., Pevzner D.V., Pogosova N.V., Protopopov A.V., Skrypnik D.V., Tereshchenko S.N., Ustyugov S.A., Khripun A.V., Shalaev S.V., Shpektor V.A., Yakushin S.S. (2021). 2020 Clinical practice guidelines for acute coronary syndrome without ST segment elevation.. Russian Journal of Cardiology.

[2] Byrne R.A., Rossello X., Coughlan J.J., Barbato E., Berry C., Chieffo A., Claeys M.J., Dan G.A., Dweck M.R., Galbraith M., Gilard M., Hinterbuchner L., Jankowska E.A., Jüni P., Kimura T., Kunadian V., Leosdottir M., Lorusso R., Pedretti R.F.E., Rigopoulos A.G., Rubini Gimenez M., Thiele H., Vranckx P., Wassmann S., Wenger N.K., Ibanez B., ESC Scientific Document Group. (2023). 2023 ESC Guidelines for the management of acute coronary syndromes.. Eur Heart J.

[3] Tsivanyuk M.M., Geltser B.I., Shakhgeldyan K.I., Emtseva E.D., Zavalin G.S., Shekunova O.I. (2022). Electrocardiographic, echocardiographic and lipid parameters in predicting obstructive coronary artery disease in patients with non-ST elevation acute coronary syndrome.. Russian Journal of Cardiology.

[4] Kuznetsova K.V., Bikbaeva G.R., Sukhinina E.M., Taumova G.Kh., Benyan A.S., Duplyakov D.V., Tukhbatova A.A., Adonina E.V., Kislukhin T.V., Nagornova V.V. (2024). Computed tomography angiography or invasive coronary angiography in patients with lowto intermediate risk acute coronary syndrome — a single-center study.. Russian Journal of Cardiology.

[5] Nesova A.K., Ryabov V.V. (2024). Paradoxes of non-ST-segment elevation acute coronary in real-life clinical practice settings.. Russian Journal of Cardiology.

[6] Kabiri A., Gharin P., Forouzannia S.A., Ahmadzadeh K., Miri R., Yousefifard M. (2023). HEART versus GRACE score in predicting the outcomes of patients with acute coronary syndrome; a systematic review and metaanalysis.. Arch Acad Emerg Med.

[7] Zykov M.V., D’yachenko N.V., Trubnikova O.A., Erlih A.D., Kashtalap V.V., Barbarash O.L. (2020). Comorbidity and gender of patients at risk of hospital mortality after emergency percutaneous coronary intervention.. Kardiologiia.

[8] Tsivanyuk M.M., Geltser B.I., Shakhgeldyan K.I., Vishnevskiy A.A., Shekunova O.I. (2022). Parameters of complete blood count, lipid profile and their ratios in predicting obstructive coronary artery disease in patients with nonST elevation acute coronary syndrome.. Russian Journal of Cardiology.

[9] Geltser B.I., Tsivanyuk M.M., Shakhgeldyan K.I., Emtseva E.D., Vishnevskiy A.A. (2021). Cardiometabolic risk factors in predicting obstructive coronary artery disease in patients with non-ST-segment elevation acute coronary syndrome.. Russian Journal of Cardiology.

[10] Namazi G., Heidar Beygi S., Vahidi M.H., Asa P., Bahmani F., Mafi A., Raygan F. (2023). Relationship between red cell distribution width and oxidative stress indexes in patients with coronary artery disease.. Rep Biochem Mol Biol.

[11] Chaulin A.M., Grigorieva Yu.V., Pavlova T.V., Duplyakov D.V. (2020). Diagnostic significance of complete blood count in cardiovascular patients.. Russian Journal of Cardiology.

[12] Nagula P., Karumuri S., Otikunta A.N., Yerrabandi S.R.V. (2017). Correlation of red blood cell distribution width with the severity of coronary artery disease — a single center study.. Indian Heart J.

[13] Zhao Z., Zhang X., Sun T., Huang X., Ma M., Yang S., Zhou Y. (2024). Prognostic value of systemic immune-inflammation index in CAD patients: systematic review and metaanalyses.. Eur J Clin Invest.

[14] Choi D.H., Kang S.H., Song H. (2016). Mean platelet volume: a potential biomarker of the risk and prognosis of heart disease.. Korean J Intern Med.

[15] Bekler A., Ozkan M.T., Tenekecioglu E., Gazi E., Yener A.U., Temiz A., Altun B., Barutcu A., Erbag G., Binnetoglu E. (2015). Increased platelet distribution width is associated with severity of coronary artery disease in patients with acute coronary syndrome.. Angiology.

[16] Vogiatzis I., Samaras A., Grigoriadis S., Sdogkos E., Koutsampasopoulos K., Bostanitis I. (2019). The mean platelet volume in the prognosis of coronary artery disease severity and risk stratification of acute coronary syndromes.. Med Arch.

[17] Liou K., Negishi K., Ho S., Russell E.A., Cranney G., Ooi S.Y. (2016). Detection of obstructive coronary artery disease using peak systolic global longitudinal strain derived by twodimensional speckle-tracking: a systematic review and meta-analysis.. J Am Soc Echocardiogr.

[18] Sharma S., Lassen M.C.H., Nielsen A.B., Skaarup K.G., Biering-Sørensen T. (2023). The clinical application of longitudinal layer specific strain as a diagnostic and prognostic instrument in ischemic heart diseases: a systematic review and meta-analysis.. Front Cardiovasc Med.

